# A Case of Cerebro-Spinal Anæmia

**Published:** 1867-01

**Authors:** R. C. Hamill

**Affiliations:** Consulting Physician to the County Hospital, Chicago


### SELECTIONS.

---

*A Case of Cerebro-Spinal Anæmia.* By R. C. Hamill, M. D., Consulting Physician to the County Hospital, Chicago.

I was called to see Miss L., a girl in her thirteenth year, October 14th, 1865, and found her dressed, lying on the bed, apparently in good health. Her mother informed me that on the day previous, on returning from school, she complained of unusual weariness and weakness of the back, but no especial attention was given to it. On the following morning, when she attempted to get out of bed, she found herself unable to stand. In this condition I found her during the afternoon of that day.

Her case presented the following phenomena: Constant, but not severe pain in the head, located, according to her description, in the line of the great longitudinal fissure separating the hemispheres of the cerebrum—the pupils of both eyes were dilated to more than one-fourth of an inch in diameter. The posterior and central portions of the tongue were covered with a closely adherent grayish fur. Appetite bad for a week, and her bowels inactive rather than constipated. Skin and kidneys approximately normal in action. A subsequent analysis detected no albumen in the urine—a slight excess of acid was apparent. The action of the heart was marked by slight irregularity, but there was no suspicion of any organic disease—pulse 110 at the wrist, and soft. Inspiration 30 per minute, and irregular, evidently the result of a habit, contracted for some unknown reason—for under my direction she could breathe naturally, at the rate of 18 inspirations per minute, and with entire ease to herself. There was some tenderness along the whole course of the spine, with marked soreness in the lower cervical and upper two dorsal vertebræ, and in the lumbar region, extending as high as the last dorsal vertebræ, inclusive. There was no obliquity of the spinal column. Sensation was perfect everywhere, and she had complete use of her limbs, turning and rolling in bed without pain and with apparently as perfect ease and freedom as if in the enjoyment of full strength and vigor. It was only when she assumed the erect position that any want of control or modification of motion could be observed on the lower limbs. She was unable to take a single step, unless the entire weight of the body was supported by the hands of attendants in the axilla of either side, and then the feet were rather jerked forward a few inches at a time, and in a manner such as to convey the impression that she was bending the entire energy of her will to the effort. The pelvis was thrown forward, the shoulders backward, and if not supported would invariably fall backwards. About two years prior to this time she had a severe attack of rheumatic fever, but I was unable to learn anything definite in relation to its history. She had on several occasions suffered from ascarides, but not within the last six months. I was inclined to attribute the loss of power to functional derangement, due to verminous irritation, and in accordance with that opinion, ordered: ℞ Santonin, grs. xii., div. chart. 4—one every four hours, to be followed by an aloetic pill. The bowels were freely moved, but no worms appeared. Infusion of quassia, ℥ iv. per anum, on the next day was followed with the same negative result, and the verminous origin of the trouble was extinguished. For the purpose of stimulating the secretions, more especially that of the liver, the following pill was ordered once in twelve hours: ℞ Pil. hydrarg. grs. xij., ext. hyosciami, grs. vi., ft. pill 6. Bowels moved once a day under the influence of the pills, and on the morning of the 18th, her tongue was quite clean, and she had some appetite for breakfast, but without other change in the case. Her pulse was soft and full, 110 to the minute; no febrile excitement; pupils dilated; pain in the head same. As a general nerve tonic and sedative, I ordered the following mixture: ℞ Citrate of iron and strychnine, ʒ i., chloric ether, ʒ ii., infus. genitan, ℥ xii., one tablespoonful three times a day, after meals, which was continued until the 1st of Nov., accompanied by thorough frictions night and morning with a soft towel, but with no perceptible benefit, as far as locomotion was involved; on the contrary, she was more helpless than at the commencement of the treatment. The iodide of potash was now substituted for the strychnine mixt., grs. vi., three times a day, frictions continued. Nov. 10th complains of a sharp pain between the left mamma and along the intercostal spaces, for which tinct. actæa racemosa, gtt. xxx. three times a day, was ordered before meals; the iodide continued two hours after meals. She remained under this treatment to the 29th; pain partially relieved, pulse varying from 100 to 110. The improvement in the main features of the case was scarcely perceptible, and I felt it my duty to call in professional aid. Dr. E. Andrews saw her with me, Nov. 30th, and after a careful examination of the case, gave as his opinion that the inability to walk was functional derangement, due to subacute inflammation of the membranes of the spinal chord at the origin of the lumbar nerves; that her diathesis was rheumatic; and that he should expect to find benefit from the use of colchicum in full doses until its cathartic effect was decided; then follow with full doses of actæa racemosa, and reduced quantity of colchicum. Accordingly, gtt. xx. of the rad. colchici, were given once in four hours. In about thirty-six hours free purging set in and the colchicum was discontinued. A Dover's powder was given at bed-time, and on the next day the following mixture was ordered: ℞ Tr. actæa racemosa, ʒ jss., vin. rad. colchici, ℥ ij., misce—gtt. xl., three times a day. The iodide of potash continued as before; croton oil to the spine, between the scapulæ; friction continued.

Dec. 15th. No relief. Her appetite, which was pretty good before, now began to fail. A short and constant cough, dependent on irritation of the laryngeal branch of the pneumogastric nerve, was a source of great annoyance. Her pulse was rarely below 110. The pain in her side much the same. She was surely losing strength without compensation in any other direction. This treatment discontinued, and I determined to address my efforts to the general health, and thus reach the nervous system through improved nutrition. With this purpose in view, the following mixture, in tablespoonful doses, three times a day, was ordered, at meal times: ℞ Hypophosphite sodæ, ℈ xii. chloric ether, ʒ ii., infus. gentian, ℥ viij., misce. The croton oil, for some reason, failed to produce counter-irritation, and a cantharidal plaster, 2½ by 8 inches, was applied between the shoulders, reaching as low as the fifth dorsal vertebræ; frictions over the lumbar region and limbs, with salted crash as before.

Dec. 18th. The blister drew finely, and was permitted to heal. The pain in the side was quite relieved by the blister. She is cheerful, and expressed herself as feeling much better. The short, hacking cough continues, but not so persistently; sometimes stopping for an hour or more; otherwise, without material change.

Jan. 1st, 1866. A decided improvement during the last ten days was manifested in every way; pulse 100; cough more under control; appetite good. Is able, by the aid of a chair, to go from her bed to the opposite side of the room.

A pair of crutches were procured for her, and in less than two weeks she was able to reach all parts of the house, through their aid.

Feb. 15th. She was able to stand without assistance, and take a few steps. Cough continued; pulse 94; pupils dilated; says she feels perfectly well.

The subsequent treatment, which reached to the middle of May, consisted of the hypophosphite of soda, as above, and the syrup of iodide of iron, gtt. xx., three times a day, alternated once in two weeks. General frictions once a day at least, with a salted towel, were continued until the case was dismissed. Her recovery was slow but uninterrupted from the 1st of January until the middle of May, when she was permitted to ride on horseback, and was able, so far as I could judge, to endure as much exercise, without fatigue, as is usual for young ladies of her age.—*Chicago Medical Journal.*

